# Dermoscopy of Lichen Planus Follicularis Tumidus: A Case Report With Pattern Analysis and Literature Review

**DOI:** 10.1002/ccr3.71880

**Published:** 2026-01-21

**Authors:** Vahidehsadat Azhari, Mina Saber

**Affiliations:** ^1^ Department of Dermatopathology, Razi Hospital Tehran University of Medical Sciences Tehran Iran; ^2^ Department of Dermatology, Skin Diseases and Leishmaniasis Research Center, School of Medicine Isfahan University of Medical Sciences Isfahan Iran

**Keywords:** comedo‐like structure, dermoscopy, lichen planus follicularis tumidus, milia‐like structure

## Abstract

Lichen planus follicularis tumidus (LPFT) is a rare variant of lichen planus presenting as erythematous to violaceous plaques with significant follicular involvement, most commonly in the retroauricular area. We report a case demonstrating a novel dermoscopic pattern reminiscent of a follicular galaxy, composed of variably sized comedo‐like and milia‐like structures with scaling, white halos, and rosettes on a pink‐violaceous background. This distinctive pattern may help distinguish LPFT from mimics, such as comedonal discoid lupus erythematosus, milia en plaque, and follicular mucinosis. Additionally, a review of existing literature on LPFT dermoscopy is included.

## Take‐Home Message

Lichen planus follicularis tumidus (LPFT) is a rare variant of lichen planus with characteristic follicular involvement. A newly described dermoscopic pattern, consisting of variably sized comedo‐like and milia‐like structures with white halos, rosettes, and a pink‐violaceous background, serves as a distinctive clue aiding in the differentiation of LPFT from its clinical mimickers.

## 1 Introduction

Lichen planus follicularis tumidus (LPFT) is a rare, distinct form of lichen planus characterized by swollen, erythematous to violaceous plaques with prominent follicular involvement [1]. This condition primarily affects middle‐aged women and tends to occur in the retroauricular region, although involvement of the cheeks, neck, ears, nose, and scalp has also been reported. Emerging evidence suggests potential links with Hepatitis B, Hepatitis C [2], autoimmune thyroiditis [1], and the coexistence of other lichen planus types, indicating a multifactorial origin. While the exact cause of LPFT remains unknown, current data point to a T‐cell‐mediated autoimmune reaction targeting the follicular epithelium [3]. Diagnosis is based on clinical features, dermoscopic examination, and histopathological confirmation [1]. Key histopathological features include dilated hair follicles with infundibular cystic changes, a dense perifollicular lichenoid lymphocytic infiltrate with vacuolar degeneration of the infundibular basal layer, and relative sparing of the interfollicular epidermis, which helps differentiate LPFT from other lichenoid dermatoses [3]. To date, fewer than 30 cases of LPFT have been reported, with only 3 including dermoscopic findings [4]. Reported dermoscopic features encompass violaceous structureless areas with fine adherent scales, Wickham's striae, and multiple white dots resembling comedone‐like openings and milia‐like cysts [1]. Additional features include perifollicular pigmentation, follicular plugs, rosettes, and an irregular brownish‐blue pigment network [5, 6]. Although not fully studied, these patterns offer valuable diagnostic clues for distinguishing LPFT from other lichenoid dermatoses. In this report, we present a new LPFT case involving the right preauricular region, with detailed dermoscopic documentation, and introduce the new dermoscopic pattern resembling a follicular galaxy. We also provide a comprehensive review of existing literature on LPFT dermoscopy to improve diagnostic recognition of this rare condition and analyze dermoscopic differential diagnoses.

## 2 Case History and Examination

**FIGURE 1:**
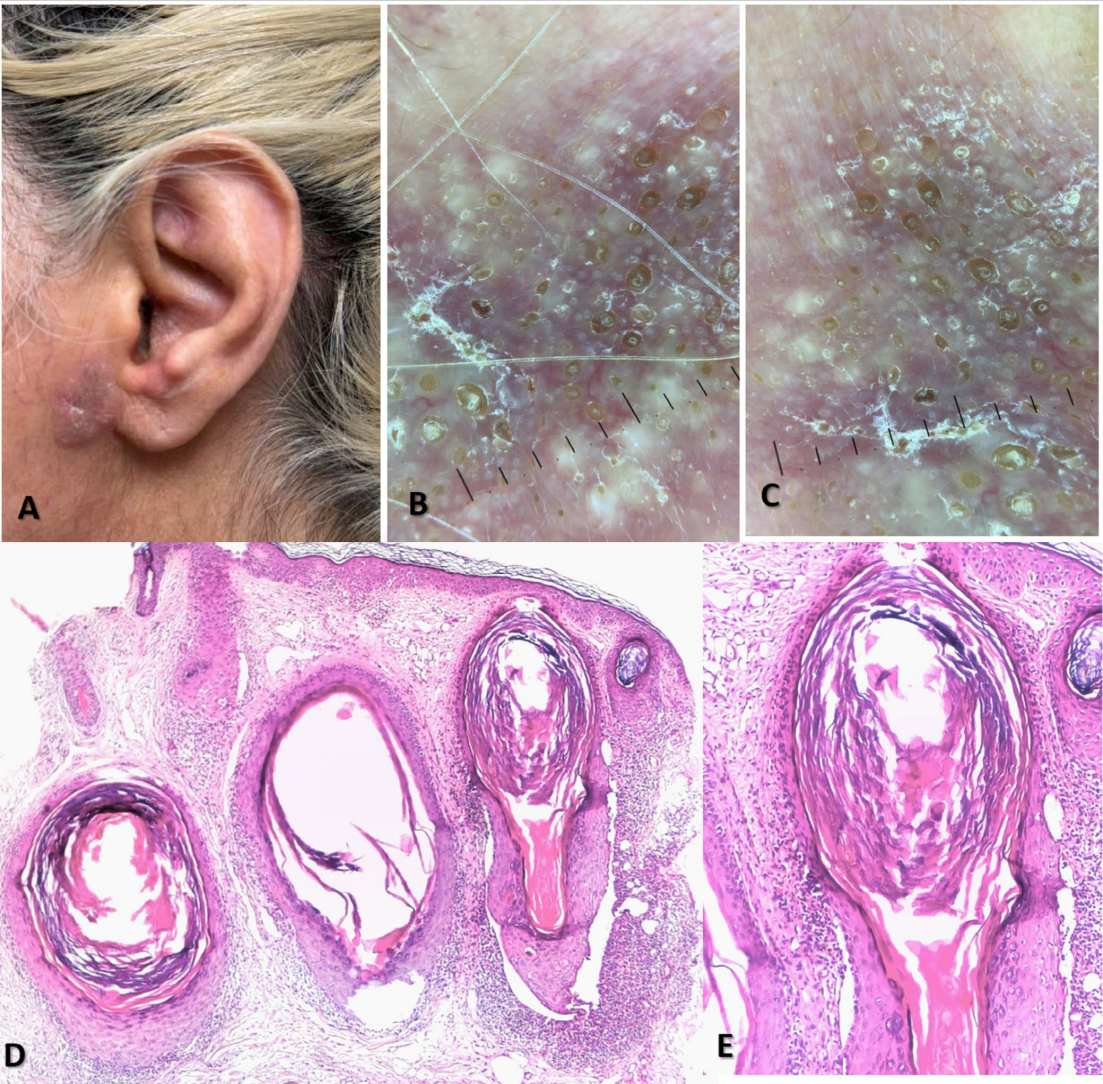
A 45‐year‐old woman presented with an erythematous‐brownish, indurated plaque in the preauricular region (A). Dermoscopy revealed multiple, variably‐sized comedo‐like and milia‐like structures, along with interspersed small rosettes on a pink‐violaceous background (B, C). Histopathological examination demonstrated cystic dilation of the follicular infundibulum and a prominent perifollicular lichenoid lymphocytic infiltrate, with notable sparing of the interfollicular epidermis (D, E).

A 45‐year‐old woman with no significant health issues presented with a one‐year history of an asymptomatic, mildly itchy, erythematous‐brownish indurated plaque located on the right preauricular area below the tragus (Figure 1A). The patient's medical history was unremarkable. Routine laboratory tests, including liver function tests and thyroid studies, were within normal limits. Dermoscopic examination revealed multiple variable‐sized comedo‐like and milia‐like structures with peripheral fine scaling; some of the comedo‐like structures had a white halo around the edges. Small rosettes were seen interspersed among the comedo‐like and milia‐like structures in a pink‐violaceous background. This dermoscopic pattern resembled a follicular galaxy (Figure 1B,C). Histopathology revealed cystic dilation of the follicular infundibulum, a prominent perifollicular lichenoid lymphocytic infiltrate, vacuolar degeneration of basal keratinocytes, and pigment incontinence. Importantly, the interfollicular epidermis was spared (Figure 1D,E). Written informed consent was obtained from the patient for publication of this case report and accompanying clinical, dermoscopic, and pathologic images.

## 3 Differential Diagnosis, Investigations, and Treatment

The main conditions to distinguish from LPFT are Comedonal Discoid Lupus Erythematosus (shows interfollicular inflammation), Milia en Plaque (uniform milia, less inflammation), and Follicular Mucinosis (uniform follicles with mucin halos). Options of treatment include topical/intralesional corticosteroids, oral retinoids, or systemic agents for refractory cases. However, this patient declined treatment.

## 4 Conclusion and Results

LPFT has a unique dermoscopic pattern, including variably sized comedones/milia with halos and rosettes on a pink‐violaceous background. This pattern is a key diagnostic clue, though histopathology remains definitive. The diagnosis for this patient was confirmed clinically, dermoscopically, and histopathologically. The asymptomatic patient opted for monitoring without treatment. At the 3‐month follow‐up, the plaque was stable.

## 5 Discussion

Lichen planus follicularis tumidus was first described in 1977 by Belaïch et al., who reported three cases of retroauricular LPFT in conjunction with otherwise typical LP at other skin sites [7]. To date, fewer than 30 cases of LPFT have been documented in the literature, most involving the retroauricular area [1]. Clinically, it appears as red‐to‐violaceous plaques, studded with comedo‐like lesions and keratin‐filled milia‐like cysts [8, 9]. Histopathologically, LPFT is characterized by cystically dilated follicular infundibula within the dermis, surrounded by a lichenoid lymphoid infiltrate and an associated interface reaction. The interfollicular epidermis is spared [3]. However, in comedonal discoid lupus erythematosus, a key differential diagnosis for LPFT, the interfollicular epidermis shows lichenoid inflammation [10]. Other clinical differential diagnoses include milia en plaque, which lacks perifollicular inflammation, and follicular mucinosis, presenting with dilated follicular infundibula and cysts filled with mucin along with a perifollicular lymphohistiocytic infiltrate [3]. Dermoscopy is an invaluable tool in diagnosing LPFT, helping distinguish it from conditions such as lupus comedonicus, milia en plaque, and follicular mucinosis, which may have similar clinical features [1]. To date, three studies [1, 5, 6] have outlined the dermoscopic pattern of LPFT, with their main findings summarized in Table 1.

**TABLE 1:** Summary of dermoscopic and histopathologic review of reported cases.

	Year	Age	Location	Dermoscopic findings	Histopathologic findings	Treatment
Hatice Kaya Ozden^6^	2017	54 Y/O female	Left retroauricular	Irregular brownish‐blue pigment network, milia‐like cysts, and comedo‐like openings	Hyperkeratosis, wedge‐shaped hypergranulosis, irregular acanthosis, and destruction of the basal layer of epidermis. Band‐like lymphohistiocytic infiltrate and prominent pigment incontinence in the upper dermis. Lymphohistiocytic inflammatory infiltrate surrounded hair follicles and cysts.	Topical clobetasol
Mariam Tabka^5^	2021	58 Y/O male	Bilateral retroauricular	Perifollicular pigmentation, follicular plugs, multiple rosettes, and Wickham striae	Slightly acanthotic epidermis with wedge‐shaped Hypergranulosis. Dilated follicular infundibulum filled with orthokeratotic keratinous lamellae, surrounded by a band‐like lymphocytic infiltrate and pigment incontinence.	Topical potent corticosteroid
Prem Prakash Pravakar^1^	2025	26 Y/O male	Left retroauricular	Violaceous structureless area with fine adherent scales, Wickham's striae and multiple white dots resembling comedo‐like openings	Orthokeratotic hyperkeratosis with focal parakeratosis, follicular plugs, and a few keratin cysts intraepidermally. The dermis showed interface changes, particularly in relation to the infundibular epithelia. Dense aggregates of lymphocytes in the dermis, and melanophages in the upper dermis.	Topical potent corticosteroid
Current study	2025	45 Y/O female	Right preauricular	Variable‐sized milia‐like and comedo‐like structures, multiple rosettes, peri‐comedo‐like white halo, pink‐purple background (Follicular galaxy sign)	Cystic dilation of the follicular infundibulum with a prominent perifollicular lichenoid lymphocytic infiltrate, vacuolar degeneration of basal keratinocytes, and pigment incontinence. Sparing of the interfollicular epidermis.	Topical potent corticosteroid

**FIGURE 2:**
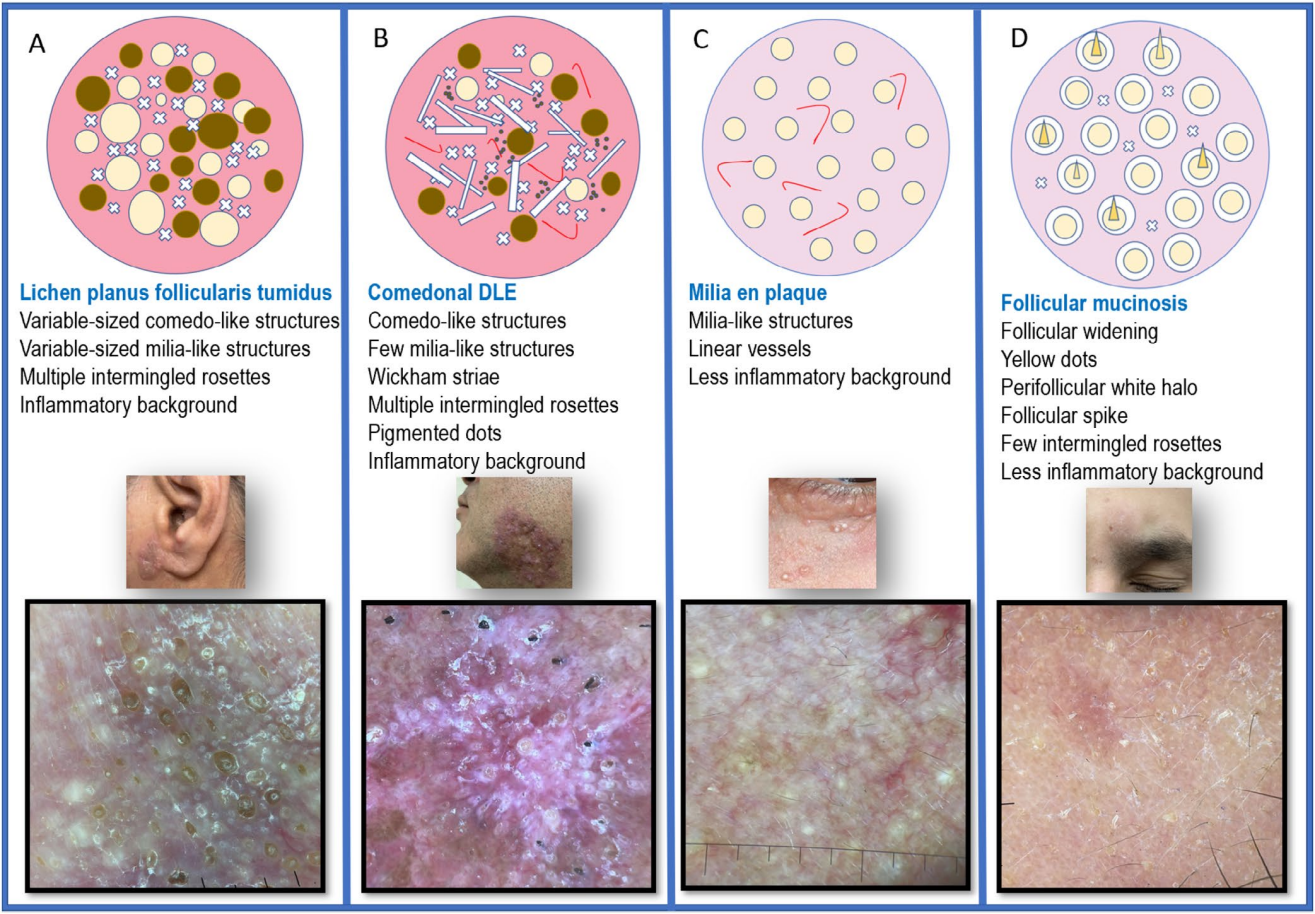
Dermoscopic patterns in disorders with clustered follicular dilation. Panels A–D illustrate findings in lichen planus follicularis tumidus, comedonal discoid lupus erythematosus, milia en plaque, and follicular mucinosis, respectively. The figure includes a schematic overview and detailed dermoscopic features.

In the current case, comedon‐like and milia‐like structures correspond to dilated hair follicles and infundibular cysts. The size variation indicates different degrees of follicular dilation and cystic expansion. Intermingled rosettes relate to remaining hair follicles with intrafollicular concentric keratin. The white halo around some comedones represents the dilated infundibular segment extending into the deeper layers, filled with keratin. The background appears diffusely pink to violaceous, indicating dermal inflammation. These dermoscopic features create a distinctive pattern reminiscent of a star cluster, called the “follicular galaxy pattern.” While this pattern suggests LPFT in the appropriate clinical context, it can also be seen in other disorders characterized by clustered follicular dilations, making clinicopathological correlation essential for a definitive diagnosis. Although comedonal discoid lupus erythematosus (DLE) can display all the dermoscopic features of LPFT, features such as interfollicular epidermal lichenoid changes causing Wickham's striae, pigmentary changes, and telangiectasia help differentiate [11]. In milia en plaque, the milia are generally uniform in size, with only a few scattered comedo‐like structures. Its background appears less inflamed and more skin‐colored [12]. Follicular mucinosis shows uniformly sized follicular dilations with a perifollicular white halo due to mucin deposition [13]. Key dermoscopic distinctions from other differential diagnoses are summarized in Figure 2. In conclusion, we propose a novel dermoscopic pattern indicative of follicular involvement as a diagnostic clue for cutaneous disorders presenting with clustered follicular dilation, such as LPFT. Given its rarity, further case reports and dermoscopic studies are warranted to refine diagnostic criteria and enhance the recognition of this condition.

## Author Contributions

**Vahidehsadat Azhari:** conceptualization, formal analysis, funding acquisition, project administration, resources, validation, writing – review and editing. **Mina Saber:** conceptualization, data curation, funding acquisition, methodology, writing – original draft, writing – review and editing.

## Funding

The authors have nothing to report.

## Ethics Statement

This study was conducted in accordance with the principles outlined in the Declaration of Helsinki of the World Medical Association. It was approved by the Ethics Committee of the Isfahan University of Medical Sciences (approval code: IR.MUI.MED.REC.1404.170).

## Consent

Written informed consent was obtained from the patient for the publication of this case report and accompanying images.

## Conflicts of Interest

The authors declare no conflicts of interest.

## Data Availability

All data supporting the findings of this case report are included in this article. Further details are available from the corresponding author upon reasonable request.
